# Holo-omics for deciphering plant-microbiome interactions

**DOI:** 10.1186/s40168-021-01014-z

**Published:** 2021-03-24

**Authors:** Ling Xu, Grady Pierroz, Heidi M.-L. Wipf, Cheng Gao, John W. Taylor, Peggy G. Lemaux, Devin Coleman-Derr

**Affiliations:** 1 Department of Plant and Microbial Biology, University of California, Berkeley, CA USA; 2 Plant Gene Expression Center, USDA-ARS, Albany, CA USA

## Abstract

Host-microbiome interactions are recognized for their importance to host health. An improved understanding of the molecular underpinnings of host-microbiome relationships will advance our capacity to accurately predict host fitness and manipulate interaction outcomes. Within the plant microbiome research field, unlocking the functional relationships between plants and their microbial partners is the next step to effectively using the microbiome to improve plant fitness. We propose that strategies that pair host and microbial datasets—referred to here as holo-omics—provide a powerful approach for hypothesis development and advancement in this area. We discuss several experimental design considerations and present a case study to highlight the potential for holo-omics to generate a more holistic perspective of molecular networks within the plant microbiome system. In addition, we discuss the biggest challenges for conducting holo-omics studies; specifically, the lack of vetted analytical frameworks, publicly available tools, and required technical expertise to process and integrate heterogeneous data. Finally, we conclude with a perspective on appropriate use-cases for holo-omics studies, the need for downstream validation, and new experimental techniques that hold promise for the plant microbiome research field. We argue that utilizing a holo-omics approach to characterize host-microbiome interactions can provide important opportunities for broadening system-level understandings and significantly inform microbial approaches to improving host health and fitness.

Video abstract

**Supplementary Information:**

The online version contains supplementary material available at 10.1186/s40168-021-01014-z.

## Introduction

The host microbiome has emerged as a crucial determinant of host health and an important modulator of host interaction with its abiotic environment [1]. In effect, the microbial genes in the microbiome augment the host’s own genetic repertoire and can act to improve the host’s adaptation to environmental perturbation [2], or, in some cases, prevent it from doing so [3]. In humans, it is increasingly recognized that the microbiome can influence a wide range of pathologies, including cancer, cardio-metabolic diseases, allergies, and obesity [4]. Similarly, it is now recognized that much of plant fitness depends on interactions with the plant microbiome [5], which not only includes susceptibility to diseases [6] but also survivability under both biotic and abiotic stress [7, 8]. Recent research suggests that these interactions depend on complex molecular exchanges involving the host’s perception of its microbial partners, microbial perception of the host, and microeconomics revolving around nutrients and resources important for survival of both [9–13]. Additionally, new findings indicate that final outcomes for host fitness can be dependent not only on the exchange of goods between the host and microbe, but on signaling and metabolic interactions among members of the microbiome themselves [14–16]. Collectively, these studies demonstrate that understanding the plant microbiome will likely require an examination of these relationships at the level of functional capacity, activity, and molecular exchange for both host and microbe.

Currently, we lack this necessary functional insight into plant microbiome interactions. This is in part due to the complexity of the system. Unlike the animal gut, the plant microbiome is assembled from and resides within one of the most diverse surrounding environments on the planet, the soil itself [1, 17]. Soils harbor a vast microbial ecosystem including bacteria, viruses, fungi, archaea, and protists, which all interact with each other [18] in complex trophic exchange networks. These soil microbiomes can shift drastically in abundance, composition, and activity over short physical distances, timeframes, and in response to seasonal environmental factors, which increases the source diversity from which plants draw their microbiomes [19–21]. As an added layer of complexity, it is also known that metabolites present within and exuded by the plant feed the microbiome [22], and that these metabolites can shift dramatically in composition and quantity over the course of plant development and from tissue to tissue, leading to differential recruitment of microbial taxa across time and space. Furthermore, specific microbial lineages are known to trigger the systemic exudation of specific plant metabolites [23], potentially creating feed forward loops in microbiome development. Collectively, these factors produce a dynamic and interconnected biological system that has challenged our ability to decipher the basal molecular mechanisms that create and sustain it.

However, perhaps a greater cause of our slow rise to functional insight on plant microbiome interactions lies in our choice of tools. The field of plant microbiome research thus far has largely relied on descriptive investigations of community structure using amplicon-based sequencing, such as 16S rRNA sequencing for bacteria and ITS sequencing for fungi [19, 20]. While it is true that these data have led to considerable insight into the general forces that act to shape the broad structure of the plant microbiome and the relative strength of their impact [24, 25], they typically fall short of providing mechanistic insight into relationships with the host. More recently, a greater number of studies have begun to explore other microbiome features, such as activity and functional capacity, through the inclusion of metatranscriptomics and shotgun metagenomics [26, 27]. Despite this increase, at present there remains a shortage of studies which take the additional step of linking plant microbiome data to plant physiology, genetics, metabolism, and other host processes [28], which could provide missing data from this underrepresented side of plant-microbiome interactions.

To achieve a more integrated perspective on plant microbiome function, we argue for experimental designs which pair host-centered omic strategies, such as transcriptomics, metabolomics, epigenomics, and proteomics, with the more commonly used microbial-focused techniques, such as amplicon sequencing, shotgun metagenomic, metatranscriptomics, and exometabolomics. Nyholm et al. recently coined the phrase “holo-omics” to describe such experiments that incorporate data across multiple omic levels from both host and microbiota domains [29]. We propose that such holo-omic studies have the power to resolve the functionality of a plant microbiome ecosystem by generating an image of what is being expressed, translated, and produced during plant-microbiome interactions [19]. This multifaceted image can help winnow results obtained from each individual dataset to meaningful biological signals, and to help build support for specific hypotheses with data gathered through orthogonal approaches. In this review, we build upon the conceptual framework introduced by Nyholm et al. with a specific exploration of holo-omics in the field of plant microbiome research. We first discuss experimental design considerations for plant holo-omics studies, focusing on the value of including longitudinal designs and careful consideration of sampling strategy. Next, we present a recent case study of plant holo-omics that investigates the interaction between drought stress and the development of the sorghum microbiome, followed by several other recent examples of holo-omic studies targeting the plant microbiome. Finally, we summarize current challenges in the analysis of holo-omics datasets and explore newly developed tools for analyzing holo-omics datasets, concluding with our perspective on the importance of downstream validation and the future of holo-omics in plant microbiome research.

### Experimental design of holo-omics studies

**Fig. 1:**
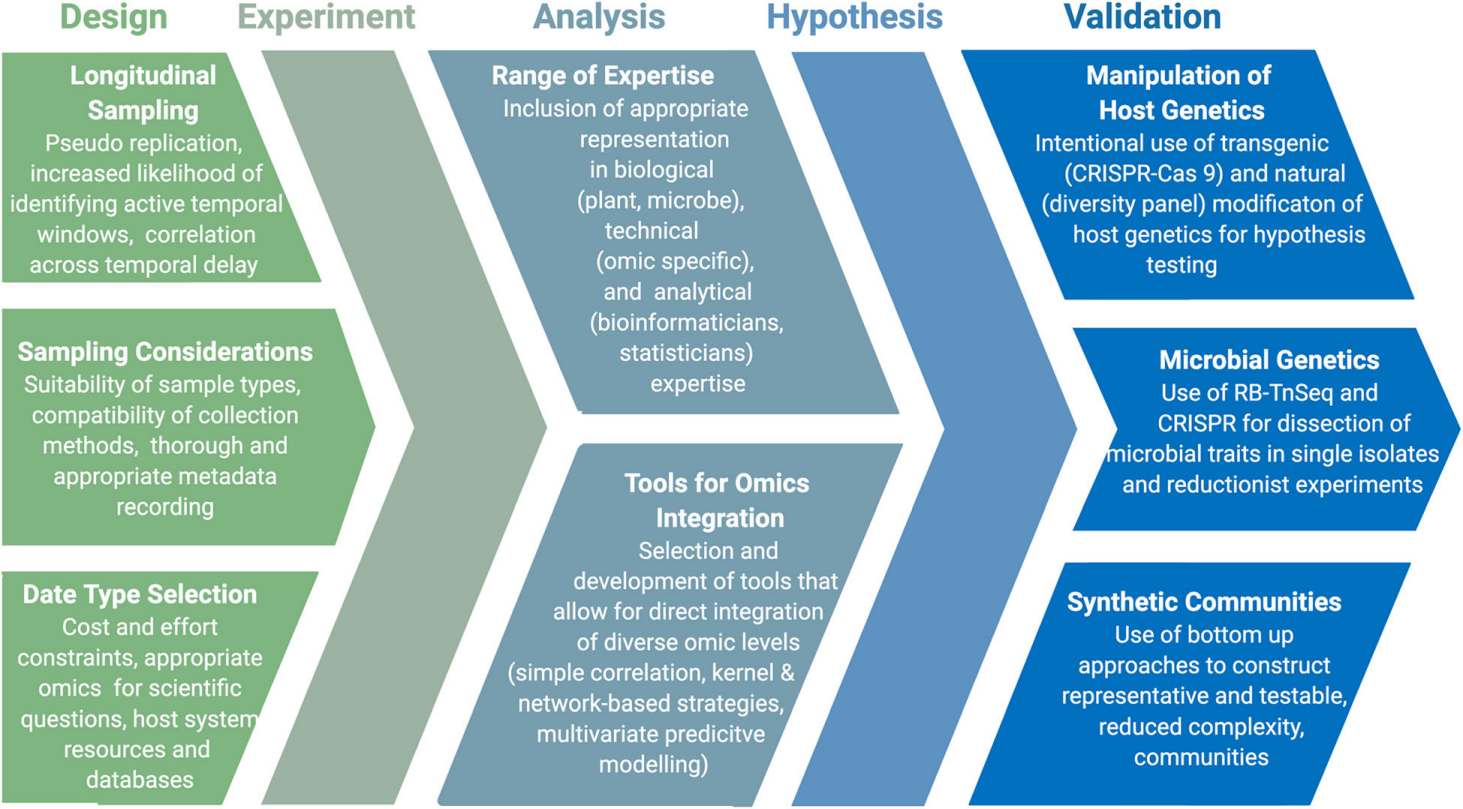
Considerations for design, analysis, and validation of holo-omic experiments. At left, design related considerations include intentional use of longitudinal designs, appropriate selection of sample types, and evaluation of optimal data types for the scientific questions addressed in the study. In the middle, analysis related challenges include selecting the appropriate range of biological and technical expertise, as well as the selection of appropriate analytical framework and tools for direct integration of diverse data types. At right, recommendations of techniques for downstream hypothesis testing and validation include use of direct and evolution-driven modifications of host genetic space, direct manipulation of microbial genetics, and bottom-up construction of reduced complexity synthetic communities

A number of experimental design considerations (Fig. 1) are crucial for obtaining accurate and meaningful results from microbiome studies that involve holo-omics [29–31]. First, we propose that longitudinal studies—used here to refer to studies in which sampling occurs across plant development, though not always from the same individual host—offer distinct advantages for holo-omic studies over their end-point counterparts. In end-point microbiome experiments, researchers’ sample at typically a single defined time point in the experiment to find differences in microbial communities between different experimental treatments. However, selecting the appropriate time point presents a challenge, as there is often little or no a priori knowledge of when host and microbiome responses will occur. More importantly, patterns in each data type, even when biologically connected or correlated, may not occur within a single temporal window.

Longitudinal studies, by contrast, attempt to describe shifts in the microbiome over time. As a result, longitudinal studies allow for a type of pseudo-replication, since patterns observed over multiple time points are more likely to be the result of real biological processes instead of random noise, similar to the value added and purpose-driven aspects of true biological replicates. Secondly, longitudinal designs increase the likelihood of observing shifts that only occur in a narrow window of time post treatment. As mentioned above, samples collected only at one arbitrarily defined time point may not capture important treatment-dependent differences manifesting outside the selected temporal window. Additionally, a longitudinal design can be beneficial in identifying correlations between data types that are affected by a temporal delay [7]. The transduction and decoding of signals between host and microbe can take time [32, 33], as does the lag between shifts in transcription and downstream shifts in protein synthesis and metabolite production within the host [34–36]. The impact on microbiome composition and abundance, and the development of macroscopic host phenotypes is likely even more impacted by temporal delay. Finally, a longitudinal design may help to establish a clear hypothesis for causality between correlated features in orthogonal datasets. The advent of probabilistic time series modeling and its application in holo-omic designs could prove particularly useful in this regard, though at present these tools are still under development [36, 37]. Despite its advantages, a longitudinal design for holo-omics experiments does come with important constraints and considerations. For instance, due to the necessity of multiple rounds of sampling, the timing of sample collection should be planned carefully to ensure that new confounding variables, such as circadian variations [38, 39] and abiotic factors that vary over diurnal cycles (for example, temperature and light), are not introduced into downstream statistical analyses. When sampling large numbers of samples, especially in the field, many host-associated data types, such as transcriptomics, are sensitive to circadian cycles and will require that samples be collected in as narrow a window at a fixed time of day across the experimental design.

Secondly, when designing an integrated holo-omics study, it is critical to consider the limitations imposed by the samples and sampling process. For instance, one must carefully consider the suitability of each collected sample type for specific desired data types, which in some cases may require alterations to sampling strategy to become feasible. Leaf microbiome samples, for example, will contain exponentially more DNA, RNA, and proteins derived from the plant than from the microbes, so some microbial techniques may not be feasible without plant-derived contamination removal. For environments that have low microbial biomass, such as sandy soils under drought stress, one must collect a larger number of samples for nucleic acid extraction. Soil or rhizosphere samples with high humic acid content, on the other hand, may require special reagents for humic acid removal. Additionally, different plant tissues, such as roots and leaves, may require different collection methods or require different amounts of time to collect, and these differences can have inadvertent impacts on sample viability and data outcomes, especially for omics strategies that are highly sensitive to time and temperature (i.e., transcriptomics, metatranscriptomics). When a single collected sample will be used to produce multiple data types, it is important to identify a universal sampling strategy that will work for all of them. Lastly, detailed collection and reporting of sample metadata is important to better ensure reproducibility and biological relevance of findings.

Third, it is important to consider not only how anticipated data types impact sampling needs but also what questions are best addressed with specific data types within a holo-omic design, and whether a holo-omic design is in fact advantageous. While there are indeed many queries that can be posed within this framework, careful review is needed of what data type combinations are most suitable and achievable for the system being studied, lines of investigation pursued, and resources available for the project; this includes taking inventory of tools, databases and computational resources available, extent and quality of host genome annotations, and potential impacts of biotic and abiotic parameters on data acquisition [40]. For example, holo-omics may be particularly challenging in non-model plants, in comparison to other host organisms, due to large, deficiently annotated genomes, large metabolic diversity, multiple organelles, and complex interaction networks with both symbionts and pathogens [36]. This may in some cases preclude the useful inclusion of some data types on the host side, such as transcriptomics or epigenomics.

It is worth noting that due to the inclusion of multiple omics techniques, holo-omics designs are typically quite expensive to implement. Specific omics techniques remain relatively expensive, including both shotgun metagenomics and metatranscriptomics, while others (such as amplicon analysis) can cost at one to two orders of magnitude less per sample. Prior to undertaking a holo-omic study, we suggest that focused pilot surveys with less costly techniques, or alternatively with limited sampling scope, have been performed first to determine that microbial community dynamics are significantly impacted by the experimental factors in question to warrant further holo-omic investigation. This will also allow preliminary analyses of the system to be analyzed without the need for as wide a range of technical and biological expertise. Development of staging within the holo-omic studies, in which techniques requiring greater investment are implemented later, can in the case of longitudinal designs allow for reduced resource expenditure through selection of critical time points to focus on based on less costly early datasets. However, it’s worth noting that not all data types are equally amenable to this approach; some sample types require immediate or rapid processing (RNA, metabolites), whereas others (DNA) can be stored for later use for much longer periods of time.

### A plant holo-omics case study

As a recent example of a holo-omics study of the plant microbiome [7], Xu et al. conducted a large-scale field study of sorghum and the associated root microbiome as it responds to drought stress. This work was carried out in the central valley of California, where a lack of summer rainfall and high temperatures virtually guarantees the ability to induce drought conditions during sorghum’s growing cycle without the need for rainout shelters [7, 41]. As plants respond to drought differently depending on their developmental stage [42], collection of time-series data in this drought experiment was employed to yield a more complete view of sorghum’s responses to water stress across growth stages. Such an approach also has value for exploring the plant microbiome; to our knowledge, very few longitudinal microbiome studies have been performed on crop systems in the field [7, 43], and even fewer exist in which the diversity, composition, and function of the plant microbial community is profiled alongside plant growth and development.

As part of this study, two genotypes of sorghum (RTx430 and BTx642) were grown in randomized blocks in an agricultural field at the Kearney Agricultural Research and Extension Center in Parlier, CA. Individual, randomized blocks were subjected to either drought stress or normal irrigation from the 2nd until the 8th week after seedling emergence [7, 44–46], at which point drought-stressed samples were watered again to explore the impact of renewed irrigation on host and microbiome processes. Samples were collected in a longitudinal fashion (once per week at a specific time of day) from leaf and root tissues, along with rhizosphere and bulk soil. This design allowed for the investigation of differences influenced by sample compartments (leaf, root, rhizosphere, and soil), by genotype (RTx430 and BTx642), by watering treatment (irrigation and drought), and by plant development (from seedling emergence to grain maturation) [44]. More importantly, the holo-omics approach described enabled exploration of connections between microbial and plant phenotypes across the diverse datasets.

First, an exploration of the impact of drought on root bacterial microbiome composition was undertaken. Amplicon sequencing (16S rRNA) revealed that the bacterial community in the sorghum root system strongly responds to early drought stress in a developmentally conditioned manner. Specifically, it was observed that drought delayed the normal development of the root and rhizosphere microbiome, and that this development is rapidly restored upon rewatering [7]. Notably, drought stress led to a strong enrichment of gram-positive bacteria, including Actinobacteria and Firmicutes, and lineages within the phylum Chloroflexi. Second, an exploration of microbiome transcriptional activity was used to look for potential causes on the microbial side for this broad, but clear, lineage-specific enrichment. Metatranscriptomics data from the rhizosphere revealed a strong drought-induced shift in microbial processes related to the transport and catabolism of carbohydrates, amino acids, and secondary metabolites, many of which are known to be present in the plant-produced root exudates that feed rhizosphere and root-associated microbes. By including metabolomics data derived from the host root tissue, we identified a strong overlap between drought-enriched root metabolites produced by the host and microbial transport pathways upregulated in the root microbiome during drought. An analysis of transcription levels through qPCR and RNA-Seq for several sorghum genes involved in these metabolic pathways revealed strong upregulation, demonstrating that the enriched metabolites were likely produced by the host and not the microbes themselves. Finally, genome-resolved metagenomics allowed for the development of partially complete genomic bins for many of the enriched and depleted taxa in the rhizosphere microbiome. A comparative genomics approach between these groups has demonstrated that microbes which are strongly enriched in drought stress have significantly more genes allocated to the transport and catabolism of many of the drought-enriched root metabolites. Based on the combined analysis of these individual datasets, we developed the hypothesis that drought leads to enrichment of specific microbes in the root microbiome through shifts in host exudate profiles that favor growth of these taxa due to substrate preference.

In addition to the bacterial community, this project also analyzed the impact of drought on sorghum’s fungal microbiome using ITS2 amplicon sequencing. The symbiosis between sorghum and arbuscular mycorrhizal fungi (AMF) was of particular interest [47], considering previous reports that AMF colonization improves drought resistance in certain plants, with speculation that fungal hyphae could improve water transport through the soil and that fungal symbionts are capable of altering their hosts’ stomatal conductance [48, 49]. In contrast, the sorghum root RNA-seq dataset found that while AMF community composition was not altered, AMF abundance decreased markedly, as assessed via qPCR of total fungal 18S rDNA and ITS2 amplicon sequencing to determine relative AMF abundance. Notably, the strongest, drought-induced change in transcription of host genes was the downregulation of the cluster previously identified as markers of AMF colonization. Transcription of these sorghum genes was strongly and broadly downregulated in both pre- and post-flowering drought and, in both cases, correlated closely with decreases in AM fungal abundance [45]. When irrigation was resumed following the pre-flowering drought, both AMF abundance and plant gene expression were restored to pre-drought levels. Furthermore, a variety of host datasets (metabolomics, proteomics, and RNA-Seq) revealed that photosynthesis, which produces the sugars used to sustain AMF partnerships, stalls during drought stress [45]. Researchers inferred from the individual analysis of these different datasets that sorghum, having lost photosynthetic output as a consequence of closing its stomata in response to drought, cannot make use of the mineral nutrients that it acquires from AMF and quits providing the sugars and lipids that it normally supplies to AMF in exchange for minerals. This experiment suggests that integrating host data with ITS amplicon data can offer profound and even surprising clarification to broad assumptions made about plant biology.

### Holo-omics research in the field of plant biology

**Fig. 2:**
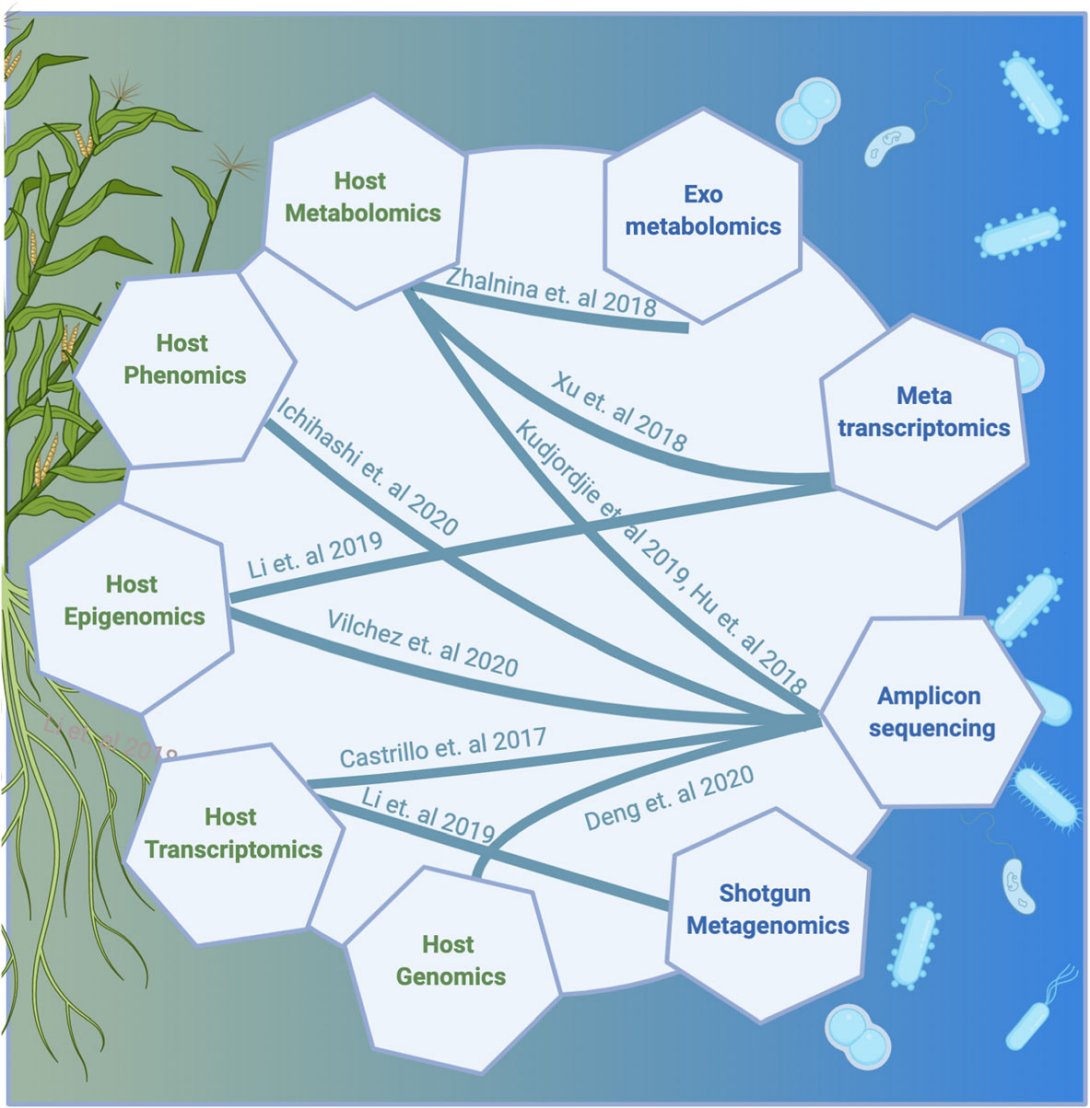
Connecting plant and microbial omics techniques. Recent examples from the plant microbiome research field of holo-omics studies employing paired datasets from host data types (in green) and associated microorganism data types (in blue). Lines between techniques indicate individual studies that integrate across the pair of indicated techniques. All studies reference here are also listed with additional detail in Table 1

In addition to the case study described above, a growing number of publications highlight the adoption of holo-omics strategies in the plant microbiome field (Table 1, Fig. 2) [31]. At present, the most frequently included host data type is transcriptomics [50–54], which affords a broad and, in many cases, comparatively well-annotated perspective on host functionality. One clear advantage of this data type is that it is perhaps the most well-developed of all the plant omics techniques in terms of analytics; there are a comparatively large number of vetted tools available, and an array of plant-host specific expression atlases for downstream analyses. As an example of one such study that successfully employed host-transcriptomics in a holo-omics framework, Castrillo et al. explored the relationship between phosphate starvation response (PSR) and microbiome composition and function in Arabidopsis [55]. As a result of this design, researchers discovered that the plant immune system coordinates microbial recognition with nutritional cues during microbiome assembly; 16S rRNA compositional profiles indicated that the microbiomes of PSR mutants were distinct from those of wild type Arabidopsis [55]. Additionally, it was shown that synthetic community inoculation enhanced the activity of a master regulator of PSR (PHR1) under limited phosphate conditions, which confirmed that PHR1 directly regulates a functionally relevant set of plant-microbe recognition genes [55]. Genome-wide gene expression analysis of the Arabidopsis root demonstrated that the PHR1 mutant in Arabidopsis also directly represses the plant immune system by altering the expression of genes in the jasmonic and salicylic acid biosynthesis pathways [55]. Taken together, this holo-omic design demonstrated that the plant root microbiome directly connects phosphate stress response and the plant immune system [55].

**Table 1:** Recent studies employing holo-omics in the field of plant microbiome research. This table lists twenty recent publications from the period of 2014 through 2020 that employ a holo-omics approach to explore plant and microbial interactions within the above or below ground plant microbiome. A subset of these studies is also indicated in Fig. 2. The first column lists the first author, year, and reference number within this review, while the second third and fourth columns indicate the plant host, sample type, and specific omics techniques employed

Refs	Plant host	Sample type(s)	Approaches
Deng et al. 2020 [61]	Sorghum	Rhizosphere	16S, host genomic
Horton et al. 2014 [62]	Arabidopsis	Leaf	16S, ITS, host genomic
Wallace et al. 2018 [63]	Maize	Leaf	16S, host genomic
Walters et al. 2018 [65]	Maize	Rhizosphere	16S, host genomic
Bergelson et al. 2019 [64]	Arabidopsis	Root	16S, host genomic
Castrillo et al. 2017 [55]	Arabidopsis	Root	16S, host RNA-seq
Zolti et al. 2020 [51]	Tomato, lettuce	Root	Shotgun metagenome, metatranscriptome, host RNA-seq
Chialva et al. 2019 [52]	Tomato	Root	Metatranscriptome, host rna-seq
Ofek-Lalzar et al. 2014 [53]	Wheat, cucumber	Root	Metagenomics, host rna-seq, metatranscriptomic
Li et al. 2019 [54]	Peanut	Root, rhizosphere	Shotgun metagenome, metatranscriptome, host RNA-seq
Kudjordjie et al. 2019 [59]	Maize	Soil, rhizosphere, root, shoot	Plant extracts, 16S
Hu et al. 2018 [58]	Maize	Root	16S, root metabolites
Huang et al. 2019 [13]	Arabidopsis, Wheat, rice and N. benthamiana	Root, leaf	Metabolites, 16S
Xu et al. 2018 [7]	Sorghum	Root, rhizosphere	Host metabolites, 16S, metatranscriptome
Varoquaux et al. 2019 [45]	Sorghum	Root, leaf	Host rna-seq, ITS
Gao et al. 2020 [44]	Sorghum	Soil, rhizosphere, root, leaf	Host rna-seq, ITS
Vílchez et al. 2020 [66]	Arabidopsis	Root	Plant and bacteria RNA quantification with qPCR, root exudates, host methylation
Ichihashi et al. 2020 [67]	Brassica rapa	Soil, rhizosphere, root	Ionomics, metabolomics, phenome, 16S
Zhalnina et al. 2018 [68]	Avena barbata	Rhizosphere	16S, isolates genomes, root exudates, exometabolomics, bacterial metabolites
Harbort et al. 2020 [15]	Arabidopsis	Root	16S, host RNA-seq
Finkel et al. 2019 [9]	Arabidopsis	Shoot, root, seedling	16S, host RNA-seq

Metabolites represent the downstream products of multiple interactions between genes, transcripts, and proteins [56] and serve as an important component of the functional interface of host-microbe interactions. An advantage of including metabolomics data in holo-omics studies is that many of the experimental, analytical, and data integration requirements that are essential for metabolomics studies are actually fully compatible with inclusion of genomics, transcriptomics and proteomics studies; for this reason, it has been suggested that metabolomics can provide a “common denominator” to the design and analysis of many holo-omics experiments [56, 57]. As an example, by combining microbiome profiling, primary metabolite quantification, host defense-gene expression, herbivore growth assays, and microbial complementation analysis, Hu et al. found that Benzoxazinoids (BXs), a class of defensive secondary metabolites that are released by the roots of cereals such as wheat and maize, create feedback loops with soil microbial communities that can alter future generations of crop performance [58]. Metabolomics was used to identify and quantify specific plant produced BX classes as they moved to soils, and amplicon analysis in several elegant experimental designs revealed that exudation of these compounds alter root-associated fungal and bacterial communities for the host and subsequent generations of the host, even following periods of overwintering. Finally, host phenomics and host gene expression analysis demonstrated that these conditioned shifts in soil microbiota led to altered host defense in the subsequent generation, including altered levels of phytohormones known to regulate herbivory [58]. An additional study in this system confirmed has suggested a role for BXs as gatekeepers to the root endosphere, allowing specific microbial lineages access to the host [59]. Despite the many advantages of using metabolomic data for holo-omic studies, some challenges remain, namely, that many peaks are not identifiable as specific metabolites and studies typically require greater replication due to noise in the data [60]. Additionally, the number of identifiable metabolites is far more limited than identifiable genes and transcripts from the genome or transcriptome layer, and as a consequence use of metabolomics data may limit the “search space” and thereby limit the interpretation of the final results [56].

Host genomic datasets have also been used recently in combination with genome-wide association style analyses to investigate the genetic underpinnings of microbial recruitment [61–65]. An analysis of a population-level microbiome analysis of the rhizospheres of 200 sorghum genotypes indicated that rhizosphere-associated bacteria exhibiting heritable associations with plant genotypes, and certain host loci showed a correlation with the abundance of specific subsets of the rhizosphere microbiome [61]. There is an opportunity to expand the use of other data types in holo-omics research as well, such as epigenomics [66], proteomics, ionomics [67], phenomics [67], exometabolomics [68], metaproteomics [69–72], and metatranscriptomics; improving analytical methodology and methods of holo-omics integration will help facilitate this expansion.

### Challenges in holo-omics analysis

Taken together, the above examples indicate how collecting a combination of microbial and host data is a promising approach to further unraveling plant and bacterial community interactions through generating mechanism-based questions and testable hypotheses. However, analysis of holo-omics comes with its own challenges (Fig. 1). One clear hurdle is that holo-omics approaches will typically require a broad range of expertise to implement, and collaborations should include not only a team of plant and microbial biologists for interpreting and decoding connections within and between kingdom-specific pathways and genes but also statisticians and computational biologists to identify and implement the approaches with appropriate statistical rigor [73]. Integrative analyses for holo-omics require intensive computational resources, including suitable means for storing, processing, analyzing data, and workflows with appropriate quality control measures and modeling selections. Another significant challenge is the current lack of fully developed analytical methodology, and there is significant need for continued development of informative and robust bioinformatic tools. For those tools that do exist, it can be difficult to know which to select, as some will be generalizable to all data types and experiments, while others will depend on the particular questions under investigation [31]. Generally, there is much more software tailored for multi-omic analysis of either the host or microbiome in isolation [36, 74–78], as opposed to tools for integrating datasets from both simultaneously [79, 80]. For instance, gNOMO is a bioinformatic pipeline that is specifically designed to process and analyze non-model organism samples of up to three meta-omics levels—metagenomics, metatranscriptomics, and metaproteomics—in an integrative manner [81], but analysis does not extend to the host.

A second challenge is the development and implementation of statistical methods that directly integrate orthogonal datasets within a single analytical framework. Currently, the majority of plant microbiome studies that employ a holo-omic design, including the examples highlighted in this study, focus on separate omic analyses first and then integrate results from seperate layers later based on the available data and prior knowledge [82, 83]. This approach, while comparatively straightforward to implement, may miss important associations among multiple omics layers [84, 85]. In this respect, the plant microbiome research community may benefit from recent advances made in the human microbiome field; a number of recent human gut holo-omics studies have begun to use direct integration of data from different omics levels, for instance through implementation of correlation analyses (such as Spearman’s rank correlation), to directly resolve microbial taxa that correlate with specific environmental or host features [86]. It has also been suggested that more recently developed strategies, such as kernel- and network-based approaches [82], as well as Network-free non-Bayesian and Network-free Bayesian [87] approaches, may more completely uncover the non-linear relationships in host-microbe interactions [88].

As an example of tools useful for a direct integration approach, the recently developed Transkingdom network (TransNet) analysis is designed to integrate and interrogate holo-omics data. TransNet allows for the construction of networks using correlations between differentially expressed elements (e.g., genes, microbes) and integration of high throughput data from different taxonomic kingdoms. In addition, TransNet analysis can be applied to integrate any “Transomics” data between, as well as within, taxonomic kingdoms. Examples of data types suitable for TransNet include miRNAs and gene expression, proteins and metabolites, bacterial and host gene expression, and methylation data [89]. A second such example is the use of multivariate predictive modeling with the Elastic Net algorithm through stacked generalization. A recent study in humans obtained samples of the immunome, transcriptome, microbiome, proteome, and metabolome simultaneously from the same patients in order to measure the ability of each dataset to predict gestational age [90]. While at present this list remains relatively short, as holo-omics research continues to grow as a field, we anticipate the development of new models, statistical and visualization tools, and methods for omics data integration and analysis that is powerful enough to understand the underlying principles that govern complex plant microbiome systems. One last, additional area of importance is the continued development of means to incorporate non-omic data with holo-omic analyses; a recent study suggest a joint modeling approach [91], in order to further advance our understandings of how the interplay of host and the microbial world impacts not only host fitness and health, but potentially broader environmental and evolutionary change as well.

## Conclusions and perspective

The crosstalk among multiple molecular layers, within and between both host and associated microbiome, cannot be properly assessed solely by a reductionist approach that analyzes individual omics layers in isolation. While holo-omics has the power to help unlock the molecular dynamics at play within the plant microbiome [29], it is worth noting that we anticipate the primary function of such large-scale holo-omics studies is to be the generation, rather than testing, of hypotheses about functional relationships in the plant microbiome. While it has been argued that null-hypothesis testing is actually an outdated method for performing ecology studies [92], to reach a functional understanding of the molecular mechanisms at play in the plant microbiome, validation experiments that follow a traditional hypothesis-driven approach will be necessary (Fig. 1) [93]. Fortunately, a wide variety of new technical approaches in both plant and microbial biology have been developed that are well suited to the purpose of hypothesis testing in the plant microbiome. The use of CRISPR/Cas9 engineering to create plant hosts altered in core functions represents one such powerful approach that has been used for validation [94]. Additionally, use of large plant germplasm collections and mapping populations has potential power to dissect genetic loci involved in the recruitment of specific microbes through microbiome-based GWAS [61–63, 95] and related approaches. On the microbial side, the use of synthetic communities to dissect microbial contributions to host phenotype is an approach that derives its power from creating microbial communities that can mimic in form and function as the native plant microbiome, but with a level of diversity that makes manipulation manageable [93]. Similarly, randomly barcoded transposon mutagenesis sequencing (RB-TnSeq) has also been shown to be capable of identifying microbial genes involved in root colonization [96], and could prove invaluable for developing more complete bacterial genome annotations as well as experimental validation of gene function. Another promising technique for in situ manipulation and study of the plant microbiome is the use of CRISPR/Cas9-derived, sequence-specific antimicrobials [97]. This environmental CRISPR/Cas9 system could be used to remove certain species or even certain alleles within a species from a complex community in order to study its effect on the plant microbiome as a whole.

In conclusion, holo-omics represents a useful tool to be used in our efforts to develop an improved understanding into the basic biology of plant-microbiome interactions. Adoption of this strategy will in turn necessitate and fuel the development of alternative sequencing-data integration analysis techniques that may have benefit outside the realm of plant biology. Finally, we believe pursuit of this path will encourage microbial and plant biologists, as well as ecologists, statisticians, and computer scientists, to work together to develop unified experimental frameworks that integrate diverse scientific perspectives. It is this process of technical and conceptual harmonization of methodologies across the scientific community that remains perhaps the greatest challenge to affording us a more holistic view of our natural world.

## Data Availability

The datasets used and/or analyzed during the current study are available from the corresponding author on reasonable request.
